# Prevalence and prognostic role of PIK3CA E545K mutation in Iranian colorectal cancer patients 

**Published:** 2019

**Authors:** Reza Ranjbar, Somayeh Mohammadpour, Amir Torshizi Esfahani, Sina Namazian, Mohammad Yaghob-Taleghani, Kaveh Baghaei, Seyed Abdolreza Mortazavi Tabatabaei, Leila Pasharavesh, Ehsan Nazemalhosseini-Mojarad

**Affiliations:** 1 *Molecular Biology Research Center, Systems Biology and Poisonings Institute, Baqiyatallah University of Medical Sciences, Tehran, Iran*; 2 *Basic and Molecular Epidemiology of Gastrointestinal Disorders Research Center, Research Institute for Gastroenterology and Liver Diseases, Shahid Beheshti University of Medical Sciences, Tehran, Iran*; 3 *Proteomics Research Center, Faculty of Paramedical Sciences, Shahid Beheshti University of Medical Sciences, Tehran, Iran*; 4 *Gastroenterology and Liver Diseases Research Center, Research Institute for Gastroenterology and Liver Diseases, Shahid Beheshti University of Medical Sciences, Tehran, Iran*

**Keywords:** PIK3CA, Mutation, Prognosis, Colorectal cancer

## Abstract

**Aim:**

This study aimed to evaluate the distribution of PIK3CA E545K mutation in Iranian CRC patients and explored its roles in disease prognosis.

**Background:**

Deregulation of the phosphoinositide 3-kinase (PI3K) pathway contributes to the progression of tumors. The p110a (PIK3CA), a catalytic subunit of PIK3, is mutated in many types of cancers. Exon 9 (E545K) is the most frequently mutated hotspot in PIK3CA in colorectal cancer (CRC). However, the prognostic role of PIK3CA E545K mutation needs to be elucidated.

**Methods:**

Tumors from 187 CRC patients were retrospectively collected from the Taleghani and Shohada Hospital, Shahid Beheshti University of Medical Sciences, Tehran, Iran, between 2010 and 2017. PIK3CA E545K status was detected in Formalin-fixed paraffin-embedded (FFPE) tissues using PCR-RFLP methods, and validated by pyrosequencing. Correlations between PIK3CA E545K mutation clinicopathological features were analyzed.

**Results:**

The frequency of PIK3CA E545K gene mutations in CRC patients was 10.7%. Significant correlations were observed in PIK3CA E545K mutation with tumor differentiation and TNM stage (p < 0.042 and p = 0.033, respectively). Kaplan–Meier analysis showed a worse prognosis in overall survival (OS) in patients with PIK3CA E545K mutation (p < 0.001). Multivariate analysis indicated that PIK3CA E545K mutation was a detrimental factor for OS (HR = 6.497, 95% CI: 2.859-14.768, p < 0.021).

**Conclusion:**

A high frequency of PIK3CA E545K mutation was detected in Iranian CRC patients. The results of the present study suggested that PIK3CA E545K mutation may be associated with poor prognosis. These findings require further confirmation via prospective studies with larger samples.

## Introduction

 Since 1995, when for the first time the critical role of the phosphatidylinositol 3-kinase (PI3K) was discovered in the cellular trafficking events in the yeast (1), many studies have been conducted to determine the role of this kinase in eukaryotic cells, especially in human cancers. This intracellular lipid kinase has a catalytic subunit p110, encoded by a gene called *PIK3CA* (2), which synthesizes phosphatidylinositol-3, 4, 5-triphosphate (PIP3) (3). PIP3 has important roles in many tyrosine kinases signaling such as AKT /mTOR, VEGFR, and EGFR. Activation of these growth factors leads to cell growth, cancer progression, and development (4). Activation of PIK3 pathway contributes to resistance to traditional chemotherapy drugs including doxorubicin, paclitaxel, tamoxifen, trastuzumab, and bevacizumab (5); therefore in many studies somatic mutation in *PIK3CA* is considered a potential prognostic and predictive biomarker (6). Most somatic mutations result in induction of PI3K signaling pathway, typically found in *PIK3CA *whose mutation hot spots are located at five sites in exons 9 and 20 (7). These mutations most frequently occur in the helical domain (hotspots E545K and E542K) or the kinase domain (hotspot H1047R) of the PIK3CA-encoded p110 (8). Studies have reported differences between exon 9 and 20 mutations with regard to their effects on survival. They noted that PIK3CA exon 20 mutations were significantly associated with poorer overall survival (9), but the prognostic role of E545K hotspot is controversial. 

The existence of a *PIK3CA* pseudogene on chromosome 22 can interfere with the detection of mutation *E545K* on exon 9 due to 98% sequence homology with this exon (10). Here, we have used Polymerase Chain Reaction-Restriction Fragment Length Polymorphism (PCR-RFLP) method for detection of a hot-spot mutation *PIK3CA *^E545K^ in exon 9. This retrospective study evaluated the prevalence of *PIK3CA *^E545K^ mutation in Iranian CRC patients and explored its roles in disease prognosis using PCR-RFLP method. 

## Methods

**Patients and specimens**

In the present study, 122 patients with colorectal cancer diagnosed between 2010 and 2017 at Taleghani Hospital and Shohada Hospital, Shahid Beheshti University of Medical Sciences, Tehran, Iran, were enrolled. 

Formalin-fixed paraffin-embedded (FFPE) tissues of the CRCs (tumor tissues and normal adjacent tissue or NAT) were reviewed by a pathologist and the optimal block was selected for the study. Eligible patients had the following inclusion criteria: (i) patients diagnosed with adenocarcinoma histologically, (ii) available clinical data and pathology report. Clinical data of the patients were retrospectively collected by a medical record review. The data included patient’s demographics (age and sex), tumor location (right-sided if the tumor was at cecum, ascending colon and transverse colon cancers and left-sided if it was at splenic flexure, descending colon and sigmoid colon as well as rectum) (11), American Joint Committee on Cancer (AJCC) TNM stage, histological differentiation, and adjuvant chemotherapy regimen. Cancer-specific overall survival (OS) was defined as the time between the initial diagnosis until the date of death. The median follow-up period was 48 (3–82) months. Patients who died of a non-CRC cause or lost to follow-up were censored. 

**Primer design and restriction enzyme selection**

**Figure 1:**
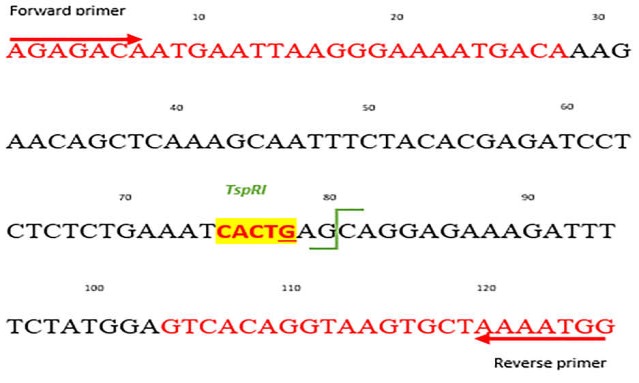
The nucleotide sequence design for the detection of the *PIK3CA E545K* mutation by PCR-RFLP. Primer sequences are highlighted in red; mutation sit G\A is underlined and ***TsprI ***recognition site is highlighted in yellow

**Figure S1:**
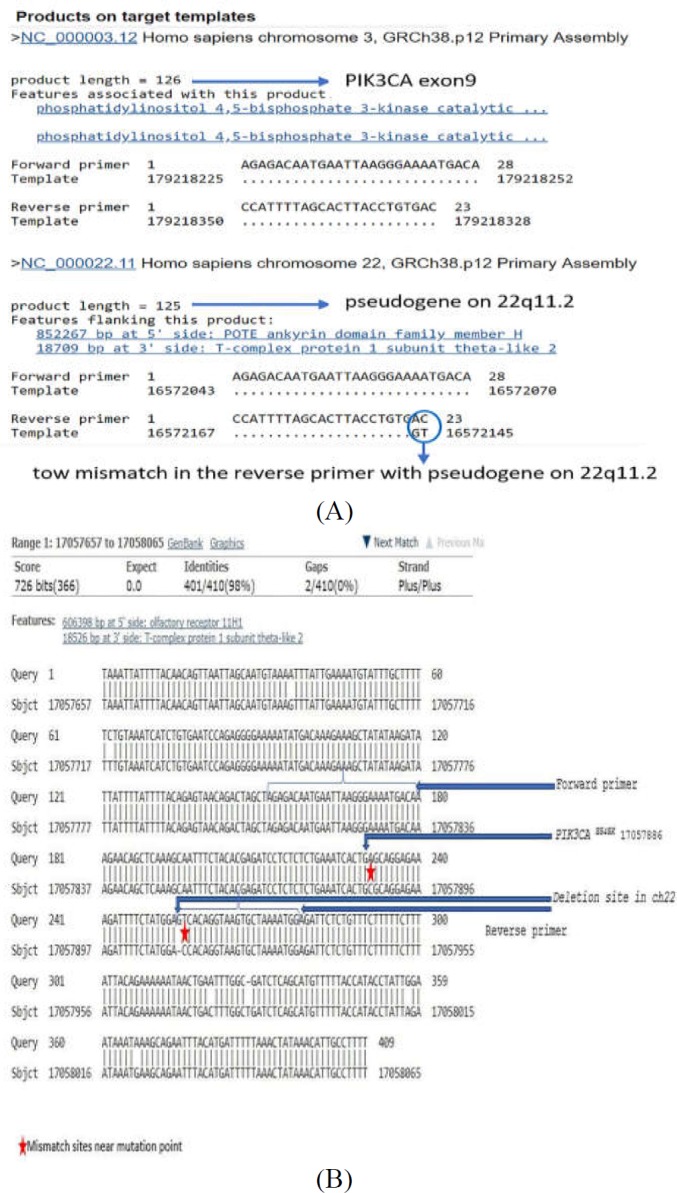
Differences between *PIK3CA* exon-9 sequence and 22q11 region. A) NCBI BLAST primer analysis result shows un-specificity of primers with tow mismatch in the reverse primer with pseudogene on 22q11.2 region. B) Deletion and mismatch sites between *PIK3CA* exon-9 sequence and 22q11 region

The information of exon 9 in *PIK3CA* gene and its mutation site rs104886003 were obtained from ENSEMBL (website http://www.ensembl.org/). A highly specific and optimum primer pair for *PIK3CA *^E545K^ was designed to avoid amplification of homologous sequences located in the chromosome 22q11.2 cateye syndrome region via Gene Runner Version 6.04 software (Table 1; supplementary Figure S1). Examination of the sequence consisting of rs104886003 for specific restriction enzyme with http://nc2.neb.com website showed that *TspRI* restriction enzyme can be employed for mutational analysis in rs104886003. The 126-bp fragment of the *PIK3CA* gene covering exon-9 sequences containing the E545K mutation and ***TspRI ***recognition site is displayed in Figure 1. In the wild-type *PIK3CA* exon-9, the 126-bp fragment could be digested into 81- and 45-bp fragments. In contrast, *PIK3CA *^E545K^ mutant alleles were not cleaved due to the substitution of CTG to CTA, resulting in the loss of the ***TsprI ***-recognized site.

**Table 1:** Primers for *PIK3CA*^ E545K^ point mutation

Primers	Method
Forward: AGAGACAATGAATTAAGGGAAAATGACA Reverse: GTCACAGGTAAGTGCTAAAATGG	PCR-RFLP
FWD: GGGAAAAATATGACAAAGAAAGCT Bio-Rev: GAGATCAGCCAAATTCAGTTAT Seq primer: TAACAGACTAGCTAGAGACA	Pyrosequencing

**Genomic DNA extraction and PCR-RFLP Experiment**

Genomic DNA (gDNA) was extracted from 10 µm sections of FFPE CRC samples using DNA FFPE Tissue Kit (QIAGEN) according to the manufacturer’s instructions. PCR was conducted in a final volume of 25 μl, containing 1 μl of the extracted genomic DNA, 2.5 μl of 10 × PCR Mg+ buffer, 1 μl of each primer, 0.5 μl dNTP, 0.3 μl of Taq DNA polymerase, and 18.7 μl double-distilled water. PCR conditions were as follows: 95˚C for 5 min; 35 cycles of 95˚C for 40 sec, 62˚C for 40 sec and 72˚C for 40 secs; and finally, 5 min at 72˚C. Electrophoresis was performed on QIAxcel® automated capillary electrophoresis system (Qiagen) via QIAxcel DNA High-Resolution Kit and QX Alignment Marker 15 bp/600 bp by OM500 separation method for 126 bp predicted PCR product. The restriction analysis of the fragments amplified with *TspRI* was performed in 2 hours at 65°C in a total reaction volume of 10 µl containing 1μl of 1× CutSmart Buffer (New England Biolabs), 0.1 μl *TspRI* enzyme (New England Biolabs), and 3.9 μl double-distilled water to digest 5 µg PCR product. Electrophoresis of the digested products was performed on the QIAxcel system with QIAxcel DNA High-Resolution Kit and QX Alignment Marker 15 bp/600 bp by OM500 separation method. *PIK3CA *^E545K^ mutation status data were analyzed by two investigators who were blinded to the patient outcomes. 

**Confirmation methods**

*PIK3CA* ^E545K^ codon and flanking sequences were amplified using the described primers based on the above PCR conditions for evaluating primer specificity. The PCR products were sequenced directly through Sanger sequencing using Big Dye Terminator v 3.1 Chemistry and an Applied Biosystems 3730 DNA Analyzer and analyzed by Finch TV Version 1.4.0. Also, to confirm the PCR-RFLP results, mutational analysis was determined by pyrosequencing. The sequencing primers are described in Table 1. Pyrosequencing was performed on the Pyro Mark Q24 (Qiagen, Germantown, MD), according to the manufacturer’s protocol. Sequence analysis was performed using PyroMark Q24 version 1.0.10 software in the allele quantification analysis mode.

**Figure 2:**
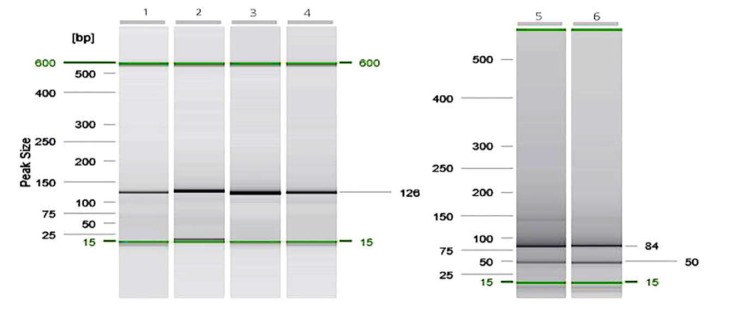
Detection of the *PIK3CA*^E545K^ mutation in CRC FFPE samples using PCR-RFLP; Lane1-4: PCR product, no-enzyme treated control, Control mutant not digested with *TspRI*, and a mutant FFPE CRC sample with a mutation on *E545K* not digested with *TspRI*, respectively (126bp). Lane 5-6: Control wild digested with *TspRI* and a wild FFPE CRC sample digested with *TspRI*, respectively (45bp and 81 bp fragments).

**Table 2:** clinicopathological features of 187 CRC patients according to *PIK3CA*^ E545K^ mutation status

	Total N (%)	*PIK3CA* ^ E545K^ status N (%)		P-value
All Patients	187	wild	mutant	
Characteristics		167 (89.3)	20 (10.7)	
Gender				
Male	111 (59.4)	102 (61.1)	9 (45.0)	0.22
Female	76 (40.6)	65 (38.9)	11 (55.0)	
Age				
<60	99 (52.9)	85 (50.9)	14 (70.0)	0.083
≥60	88 (47.1)	82 (49.1)	6 (30.0)	
Tumour Location				
Left Colon	137 (73.3)	123 (73.7)	14 (70.0)	0.45
Right Colon	50 (26.7)	44 (26.3)	6 (30.0)	
Tumour Stage				
I- II III- IV	96 (51.3) 91 (48.7)	91 (54.5) 76 (45.5)	6 (30.0) 14 (70.0)	0.033
Differentiation				
Well Moderate/ Poor	64 (34.2) 123 (65.8)	61 (36.5) 106 (63.5)	3 (15.0) 17 (85.0)	0.042
Family History				
Yes	66 (35.3)	57 (34.1)	9 (45.0)	0.23
No	121 (64.7)	110 (65.9)	11 (55.0)	

**Table 3:** Univariate and multivariate cox regression analysis of possible prognostic variables and parameters that correlate with overall survival in 187 CRC patients

Characteristics	Univariate analysis Hazard ratio for death (95% confidence interval)	*P*-value	Multivariate analysis Hazard ratio for death (95% confidence interval)	*P*-value
Age				
<60	1 ref.	0.014	1 ref.	0.198
≥60	2.215 (1.178-4.167)		1.586 (0.786-3.201)	
Gender				
Female	1 ref.	0.180	1 ref.	0.640
Male	1.557(0.815-2.974)		1.183 (0.585-2.392)	
Tumour Location				
Left Colon	1 ref.	0.014	1 ref.	0.198
Right Colon	2.215 (1.178-4.167)		1.586 (0.786-3.201)	
Tumour Stage				
II	1 ref.	0.007	1 ref.	0.356
III	2.481 (1.284-4.794)		1.474 (0.646-3.365)	
Differentiation				
Well	1 ref.	0.211	1 ref.	0.211
Poor/ Moderate	0.643 (0.322-1.284)		0.624 (0.298-1.308)	
PIK3CA ^E545K^ mutant				
Negative	1 ref.	0.001	1 ref.	0.021
Positive	3.618 (1.814-7.216)		6.497 (2.859-14.768)	

**Statistical analysis**

All data were statistically analyzed using the Statistical Package for the Social Sciences, version 21.0 (SPSS 21.0). Differences in distributions between the variables examined were assessed via the chi-square test. The survival rate in each group was analyzed using the method of Kaplan and Meier and compared by the log-rank test. The factors that affected the survival were identified using univariate and multivariate analyses with the Cox hazard regression model. Hazard ratios (HRs) with 95% confidence intervals (CIs) were reported and a *P*-value of ≤0.05 indicated a statistically significant difference. 

## Results

**Establishment of PCR-RFLP method for the detection of PIK3CA E545K**

When 126bp size fragment was applied to RFLP with TspRI enzyme, the wild and mutant-type sequences were recognizable based on the difference in the number and size of the endonuclease-digested fragment; in this regard, the wild-type fragments have TspRI recognition sites and digested by enzyme thus producing two 81 and 45 bp fragments (Figure 2, Lane5-6), while the mutant fragment does not have this recognition site so the detected fragment was 126 bp (Figure 2, Lane3-4).

**Figure S2:**
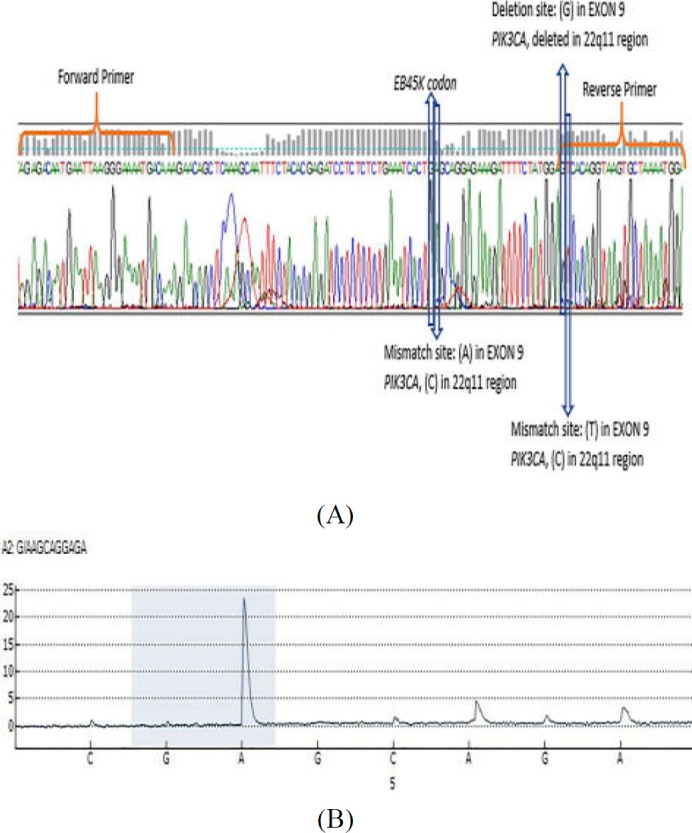
Sequencing of CRC FFPE tissue for validating the PCR-RFLP method. A) Sequencing of CRC FFPE tissue for detecting the specificity of designated primers. B) Pyrogram for amplification of a mutant sample with substitution of G with A in *PIK3CA* ^E545K^ using specific primers

Through DNA Sanger sequencing, the complete length of PIK3CA exon 9 was read and differences with pseudogene on 22q11.2 were checked (Supplementary Figure S2), and the specificity of the primers approved.

**Prevalence of PIK3CA E545K mutation and its correlation with clinicopathologic characteristics of CRC patients**

Table 2 reports the baseline characteristics of the 187 CRC patients. The frequency of PIK3CA E545K gene mutations in CRC patients was 10.7% (20/187). 

The correlation between PIK3CA E545K mutation and clinicopathologic characteristics was analyzed in Table 2. Significant correlations were observed in PIK3CA E545K mutation with tumor differentiation and TNM stage (p < 0.042 and p = 0.033, respectively). PIK3CA E545K mutation in tumor tissues was correlated with poor/moderate differentiation and late clinical stage. However, no significant correlation was found between PIK3CA E545K mutation and age, gender, or tumor location.

**Association between PIK3CA E545K mutation and survival**

**Figure 3:**
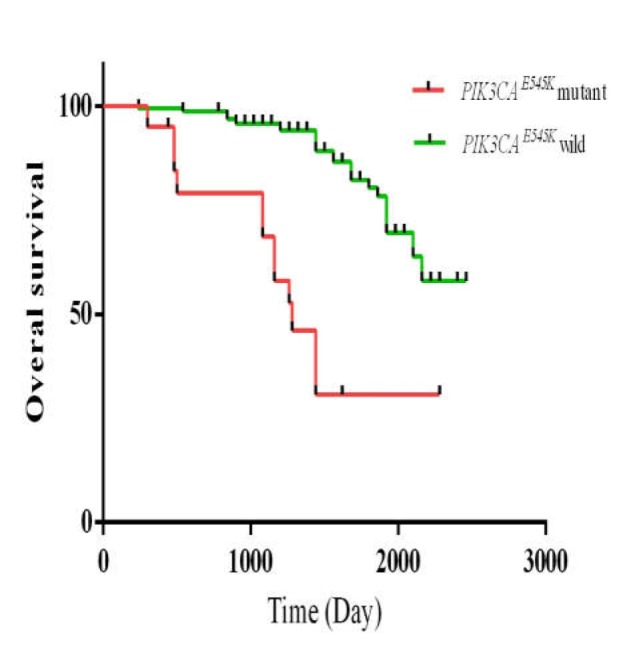
The prognostic value of *PIK3CA*^ E545K^ mutation in CRC patients; Kaplan–Meier analysis of the correlation between *PIK3CA*^ E545K^ mutation and disease-free survival of 187 CRC patients (p < 0.001)

Further analysis was performed for the prognostic significance of PIK3CA E545K mutation. Totally, 41 patients (21.9%) died by the latest follow-up. The median period for the overall survival (OS) was 42.6 months. Kaplan–Meier analysis showed a worse prognosis in OS in patients with PIK3CA E545K mutation as compared to other patients (p < 0.001; Figure 3). Univariate analysis revealed that PIK3CA E545K mutation, tumor location, and tumor stage were associated with a significantly shorter OS (Table 3). Multivariate analysis was also performed with baseline prognostic variables including age, gender, tumor location, differentiation, and PIK3CA E545K mutation indicating that PIK3CA E545K mutation was a detrimental factor for OS (HR = 6.497, 95% CI: 2.859-14.768, p < 0.021. Table 3).

## Discussion

Our study investigated the prevalence of *PIK3CA*^ E545K^ mutation in Iranian CRC patients. We provided evidence that *PIK3CA*^ E545K^ mutation was correlated with lower histological differentiation, later clinical stage, and poorer prognosis; thus, *PIK3CA*^ E545K^ mutation is a potential molecular biomarker for predicting prognosis in CRC patients.

Activating missense mutations in *PIK3CA* p110α subunit has been proven in many cancers (12). In all tumors, the most frequent *PIK3CA* mutation occurs in exon 9 (helical domain E542K and E545K) and in exon 20 (kinase domain H1047R) (13). These three most frequently reported hot spots in *PIK3CA* have been shown to elevate the lipid kinase activity of *PIK3CA* and result in the activation of its downstream Akt signaling pathway (14). They account for 80% of reported PIK3CA mutations in CRC which are also completely oncogenic in vivo (15). A plethora of literature points to the association between these mutations and resistance to common therapies especially anti-EGFR treatment as well as cancer progression (16), highlighting the need to detect PIK3CA p110α mutation as a strong predictive and prognostic biomarker. 

Numerous studies have proven that the most prevalent mutation in CRC at *PIK3CA* is E545K hot spot (17-20). In this study, exon 9 (E545K) loci were examined via PCR-RFLP. Totally, 10.7% of the CRC patients were observed with *PIK3CA *^E545K^ mutant tumors. Previous reports showed a slightly lower frequency of *PIK3CA *^E545K^ mutation which may be due to population or detection method (18-20). Pentheroudakis et al. detected the PIK3CA mutation in exon 9 in 8.8 of cases (18), while Stec et al. detected *PIK3CA *^E545K^ mutation in 7% CRCs. Prenen et al. observed *PIK3CA*^ E545K^ mutation in 5.5% CRCs (19) and De Roock found this mutation in 9.9% CRCs (20). 

Tumors with *PIK3CA *^E545K^ mutation were characterized by a predominantly distal colonic location, frequent presence of tubular differentiation and lower histological differentiation, and late clinical stage. Another study revealed that patients with *PIK3CA*^ E545K^ gene mutations were in a more advanced disease, T stage III or IV and had tumor recurrence (19). 

In 2005, Samuels et al. (21) showed in a preclinical model that *PIK3CA*^ E545K^ mutation promotes cell growth and invasion of human cancer cells. It is unclear whether other *PIK3CA* mutations have the same functional properties. E545K mutation results in an amino acid substitution of opposite charge. In this oncogenic charge-reversal mutation, the interactions between the protein catalytic and regulatory subunits are abrogated, resulting in loss of regulation and constitutive PIK3CA α subunit activity, which can lead to oncogenesis (22). However, the correlation between *PIK3CA* mutations and prognosis of CRC patients is still controversial (23). Interestingly, we found that patients with exon9 charge-plus changing substitutions in the helical domain showed even poorer survival. Although several studies have documented that mutation in *PIK3CA *^E545K^ had a negative effect on survival (9, 24), three additional studies found no significant difference between exon 9 mutants and wild-type cases in colorectal cancer-specific or overall survival (24). Note that to the best of our knowledge this is the first study reporting *PIK3CA*^ E545K^ prevalence and prognostic significance in Iranian CRCs. There are other studies regarding the most common and important molecular biomarkers as well as their prognostic roles such as BRAF and KRAS mutations (25-26) and microsatellite instability (27) in Iranian CRC patients. Further analysis is still required to assess the racial differences and the role of all-important CRC biomarkers to explore their correlations with certain clinicopathological parameters in a large cohort study.

In conclusion, our findings have been novel regarding survival in patients whose tumors harbored mutations in E545K loci of *PIK3CA* . However, given that the number of such cases in our study was small, and statistical power was consequently limited, these findings warrant validation by independent studies. Our findings might give additional insight into the relevance of the PIK3 pathway in colorectal cancer progression, and suggest that detailed genotyping of *PIK3CA* might be tailored to personalized medicine.

## Conflict of interests

The authors declare that they have no conflict of interest.

## Supplementary files
